# Morphine Suppository versus Indomethacin Suppository in the Management of Renal Colic: Randomized Clinical Trial

**DOI:** 10.1155/2016/4981585

**Published:** 2016-03-17

**Authors:** Forough Zamanian, Mohammad Jalili, Maziar Moradi-Lakeh, Maryam Kia, Rokhsareh Aghili, Seyed Mojtaba Aghili

**Affiliations:** ^1^Department of Emergency Medicine, Imam Khomeini Hospital, Tehran University of Medical Sciences, Tehran, Iran; ^2^Emergency Medicine Department, Tehran University of Medical Sciences, Tehran, Iran; ^3^Department of Community Medicine, Iran University of Medical Sciences, Tehran, Iran; ^4^Department of Internal Medicine, Dr. Ziaeian Hospital, Tehran University of Medical Sciences, Tehran, Iran; ^5^Endocrine Research Center, Institute of Endocrinology and Metabolism, Iran University of Medical Sciences, Tehran, Iran

## Abstract

*Background*. Renal colic is a medical emergency due to the rapid onset and devastating nature of its pain. Opioids and nonsteroidal anti-inflammatory drugs (NSAIDs) are both used as first-line choices in its management.* Aim*. This study aimed to compare the efficacy and safety of opioids and NSAIDs in the management of acute renal colic.* Methods*. One hundred and fifty-eight patients were divided into two groups (*n* = 79) and received either 10 mg morphine or 100 mg indomethacin suppositories. The severity of pain was measured using verbal numeric rating scale at baseline and 20, 40, 60, and 90 minutes after the administration of analgesics. Drug side effects as well as patients' vital signs were also recorded.* Results*. The mean decrease in the pain score during the first 20 minutes was significantly higher among those who received morphine suppository. However, no significant difference was observed between the two groups regarding the mean decrease in pain score during the first 40, 60, and 90 minutes after the admission. Prevalence of drug side effects or changes in the vital signs was not significantly different between the two groups.* Conclusions*. Morphine suppositories seem to be more efficient in achieving rapid pain relief comparing to indomethacin.

## 1. Introduction

Renal colic, as the most common presentation of ureteral calculi, is characterized by a sudden onset and severe sense of pain in the flanks which may radiate to the hypochondrium [1]. This pain is caused by the obstruction and the subsequent increased tension in the urinary tract during the calculi passage and is commonly compared with the delivery pain. Increased pressure of the renal pelvis provokes the secretion of prostaglandins followed by vasodilatation which results in further increase in the intrarenal pressure as a result of diuresis and smooth muscle spasm [2, 3]. Renal colic is generally considered as a serious health problem due to the devastating nature of the pain and its high prevalence in general population which results in a considerable number of lost work days. The annual incidence of renal colic is reported to be around 16 per 10,000 population with a lifetime incidence of 1–20% [4–6]. Interestingly, an increase in the incidence of ureteric calculi and renal colic has been observed during the past decades, most probably due to changes in the life style of the general population [7].

The current standard management of pain during acute renal colic episodes includes administration of opioids as well as nonsteroidal anti-inflammatory drugs (NSAIDs) both alone and in combination with each other. While opioids take advantages of being inexpensive, potent, and titratable, their usage is limited due to the fear of respiratory depression, drug dependency, and potential drug abuse by mimicking renal colic symptoms in order to receive opioids [8]. Morphine suppositories benefit from a sustained and prolonged analgesic effect which requires fewer administrations [9] and could potentially be less favorable for drug abusers who mimic renal colic. In addition, administration of morphine via rectal rout bypasses the need for intravenous catheter placement and can be given to patients in their first encounter in the triage room. Moreover, opioids have no effect on the prostaglandin secretion, as the main cause of pain in renal colic [10]. NSAIDs, on the other hand, directly inhibit the prostaglandin effect and, as a result, are probably more effective than opioids [8, 10]. However, NSAIDs may cause serious side effects, most notably renal impairment and renal failure as well as gastrointestinal bleeding [10]. Moreover, in contrast with opioids, NSAIDs are not titratable and their analgesic benefits take longer to effect [8].

Currently, the choice between opioids and NSAIDs at the clinical setting is highly dependent on clinician's personal preference and experience as well as institutional protocols while there is limited data in order to compare the outcome of these drugs in the management of acute renal colic [11]. Considering the exceedingly busy setting of emergency department and the time needed to achieve intravenous access, the current study was designed to compare the efficacy and adverse effects of the suppository administrated morphine and indomethacin used in the management of renal colic.

## 2. Methods

The current randomized double-blind clinical trial (IRCT registration ID: 2014050514297N2) was performed between March 2011 and March 2013 in the emergency department of Imam Hospital and Shariati hospital, two tertiary care university affiliated teaching hospitals. The Institutional Review Board has reviewed and approved the process of this study.

Based on the previous studies, a minimum sample size of 79 for each group was desired [12]. This was based on an alpha of 0.05, power of 0.90; and mean (*M*) and standard deviations (SD) of 24.7 and 9.1 for indomethacin and 27.4 and 9.1 for morphine, using the following formula:

(1) $$\begin{array}{c} N=\frac{{\left(Z_{1-\alpha /2}+Z_{\beta }\right)}^{2}\left({SD}_{1}^{2}+{SD}_{2}^{2}\right)}{{\left(M_{1}-M_{2}\right)}^{2}}. \end{array}$$

One hundred and fifty-eight patients with a confirmed diagnosis of renal colic who aged between 18 and 75 years were enrolled in this study and were randomly allocated into two groups using permuted-block randomization method (*n* = 79). Based on the patients' clinical presentation, the diagnosis of renal colic was primarily made by triage nurse and was then confirmed by the emergency medicine resident and the attending physician. The process of the study was explained to all participants and they were asked to fill an informed consent before being entered into the study. Patients were ensured that their personal and clinical data would not be revealed under any circumstances and their names were replaced with codes before data entry to ensure data confidentiality. The exclusion criteria were as follows: unwillingness to participate in the study or to receive suppository analgesics, pregnancy, breastfeeding, a history of current or past drug abuse, analgesic intake during up to 4 hours prior to admission, long-term use of NSAIDs, a drug history of hypnotic drugs or phenothiazines, a history of drug hypersensitivity reaction due to morphine or NSAIDs, diarrhea, peritonitis, and a history of chronic diseases including liver disorders, renal disorders, respiratory problems, gastrointestinal problems, and endocrine disorders.

Patients either received 10 mg morphine suppository (group A) or 100 mg indomethacin suppository (group B) for the management of renal colic, respectively. In order to blind the observers as well as patients, morphine and indomethacin suppositories were placed into glass containers labeled as A and B and were administrated for patients by the resident of emergency medicine. Only pharmacy section manager of hospital was aware of the type of suppositories placed into A and B containers. The primary outcome of the study was change in the pain score. The secondary outcome of the study was the safety and tolerability of drugs as measured by hemodynamic changes and adverse effects. Assessment of pain was performed using 11-point numeric rating scale (from 0 for no pain to 10 for most severe pain possible) at the time of admission and 20, 40, 60, and 90 minutes later. Intravenous morphine with a dosage of 5 mg was suggested and administrated for each patient every 20 minutes if the pain remained the same or the patient reported only a minor pain relief. Drug adverse effects including nausea, vomiting, dizziness, and drowsiness as well as patients' vital signs were also assessed every 20 minutes. Demographic data including sex and age, pain score reported by patient at various points, the amount of analgesics needed for pain relief, and the duration of hospital stay was recorded for each patient.

Data analysis was performed using SPSS software package for windows version 21 (IBM, USA). Quantitative variables are expressed as mean ± standard deviation. Repeated measure analysis and chi-squared test were used in order to compare quantitative and qualitative variables between groups, respectively. Effect size (ES) was estimated with a 95% confidence interval (95% CI). *P* values lower than 0.05 were considered as statistically significant.

## 3. Results

**Figure 1:**
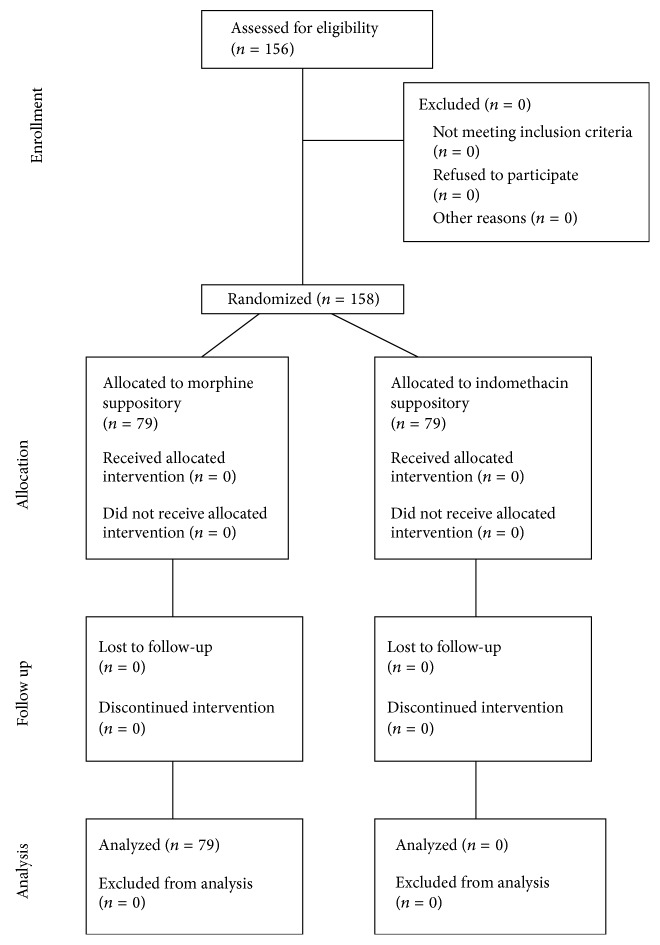
CONSORT diagram showing the flow of participants.

A total number of 158 cases including 102 (64.6%) male and 56 (35.4%) female cases were enrolled in the current study (Figure 1). The mean age of the study population was 37.4 ± 11.1 years which was not statistically different between groups A and B (37.2 ± 10.6 years versus 37.3 ± 11.5 years, *P* = 0.9, and ES = −0.01 [95% CI = −0.32 to 0.32]). The mean weight of the study population was 73.8 ± 10.4 kilograms which was not statistically different between groups A and B (74.1 ± 8.9 kilograms versus 73.4 ± 11.8 kilograms, *P* = 0.9, and ES = 0.07 [95% CI = −0.25 to 0.38]). Male to female ratio was also similar between groups A and B (1.75 versus 1.88, *P* = 0.6, and ES = 0.74 [95% CI = 0.37 to 1.10]).  Table 1 summarizes patients' demographic data.

**Figure 2:**
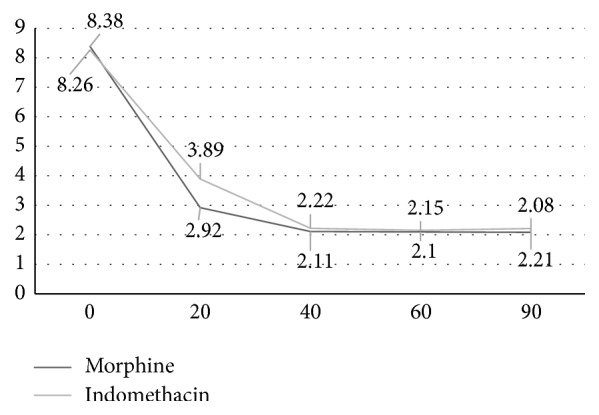
Timeline graph of pain scores in the two study groups.

The mean pain score at the start of measurements was not significantly different between the two groups (8.38 ± 1.08 for group A versus 8.26 ± 1.09 for group B, *P* = 0.5, and ES = 0.1 [95% CI = −0.22 to 0.41]). The mean decrease in the pain score during the first 20 minutes after the admission was significantly higher among group A cases who received morphine suppository comparing to group B who received indomethacin suppository (5.46 ± 1.34 versus 4.36 ± 1.62, *P* < 0.001, and ES = 0.74 [95% CI = 0.44 to 1.06]). However, no significant difference was observed between the two groups regarding the mean decrease in pain score during the first 40, 60, and 90 minutes after the admission (Table 2).  Figure 2 shows the timeline for pain scores in two study groups. The mean change in the systolic and diastolic blood pressure was not also significantly different between the two groups during the first 20, 40, 60, and 90 minutes after the admission (Table 3).

Nausea was reported by 52.7% of patients in group A and by 47.3% of patients in group B which was not significantly different (*P* = 0.5, ES = 0.11 [95% CI = −0.22 to 0.46]). Vomiting was reported by 49% of patients in group A and by 51% of patients in group B which was also not significantly different (*P* = 0.7, ES = −0.04 [95% CI = −0.38 to 0.29]). The prevalence of dizziness was 43.3% in group A and 56.7% in group B which was not significantly different (*P* = 0.4, ES = −0.29 [95% CI = −0.64 to 0.04]). Dryness of the mouth was reported by 48% of patients in group A and by 52% of patients in group B which was also not significantly different (*P* = 0.4, ES = −0.08 [95% CI = −0.43 to 0.25]). Allergic drug reactions were observed in 49.7% of patients in group A and in 50.3% of patients in group B which was also not significantly different (*P* = 0.5, EF = −0.01 [−0.35 to 0.33]).  Table 4 compares the adverse effects between the two study groups.

## 4. Discussion

According to the results of the current study, the mean pain score was not significantly different between renal colic patients who received either morphine or indomethacin suppository. However, those who received morphine experienced a greater relief in pain during the first 20 minutes after the admission. No difference was observed between these two groups regarding the drug side effects including changes in the systolic and diastolic blood pressures.

In one study, Cordell et al. investigated the differences regarding the efficacy in pain relief and probable side effects of intravenous morphine and indomethacin suppositories in patients with renal colic [10]. Their findings showed that those who received intravenous morphine achieved a better pain relief after the first ten minutes but not after 20 and 30 minutes comparing to those who received indomethacin suppository. This is in accordance with the results of this study in which morphine suppository was superior comparing to indomethacin suppository in order to achieve pain relief only during the first 20 minutes after the admission. Moreover, similar to the current study, they found no significant differences regarding the side effects of the two drugs.

Since the analgesic effects of morphine and NSAIDs in renal colic are achieved via different pathways, one may conclude that the combination of these drugs would result in a better pain management or a decrease in the amount of needed morphine as a drug with a high potential for abuse. In a study by Engeler et al. two orally administrated NSAIDs, rofecoxib and diclofenac, were used in combination with morphine in the management of acute pain of renal colic [13]. The mean morphine consumption was not significantly different among patients who received rofecoxib, diclofenac, or placebo in addition to morphine. Moreover, the pain score reported by patients was similar among these three groups. On the contrary, in a study by Safdar et al., renal colic patients who received a combination of intravenous morphine and ketorolac, NSAIDs, achieved a better pain relief with a lesser amount of medication comparing to those who received either of these two drugs alone [14]. In another study by Phillips et al. the efficacy of celecoxib, also an NSAID, in the management of renal colic was evaluated [15]. The authors reported that celecoxib was not efficient in the facilitation of the stone passage or decrease in the amount of narcotic administration was evaluated.

Several studies have focused on finding an alternative analgesic for opioids in the management of acute pain of renal colic. Aside from NSAIDs, the efficacy and probable side effects of paracetamol have also been the subject of some investigations. In one study, Bektas et al. compared the intravenous administration of morphine and paracetamol among 146 renal colic patients who visited emergency department [8]. Their findings showed that intravenously administrated morphine and paracetamol were both effective in achieving pain relief comparing to the placebo with no significant difference between the two and both drugs were well tolerated. In another study by Grissa et al. renal colic patients received either intravenous single-dose paracetamol or intramuscular single-dose piroxicam, an NSAID, after being admitted for renal colic at the emergency department [16]. The authors reported a higher rate of pain relief for paracetamol comparing to piroxicam 90 minutes after the administration of these analgesics with minimal side effects for both drugs (80% versus 48%, resp.). Moreover, the superiority in the efficacy of paracetamol comparing to piroxicam began to be apparent 45 minutes after the administration of these drugs.

The current study faces several limitations. First, some of the most serious side effects of analgesics used in this study could not be assessed due to the short period of follow-up. Drug abuse and dependence among those receiving opioids such as morphine and gastrointestinal bleeding among those receiving NSAIDs such as indomethacin are more likely to occur after patient's discharge from the emergency ward. However, it should be noted that the potential for abuse is particular for those who already have a history of drug abuse and should not be generalized to all patients after one admission. Second, it has been previously suggested that nausea and vomiting, which were expected side effects in this study, may affect patient's perception of pain by increasing the level of discomfort [14]. Finally, due to difficulties in obtaining the desired number of patients, we did not include a group receiving both morphine and indomethacin which would help to find out if the combination of these drugs could result in a better pain management and decrease in the amount of opioids administrated.

In conclusion, while the effect of analgesics on patients' vital signs and side effects of both morphine and indomethacin do not differ significantly, morphine suppositories seem to be more efficient in achieving rapid pain relief comparing to their indomethacin counterparts. Hence, morphine remains as a yet irreplaceable analgesic in the acute management of pain among renal colic patients.

## Figures and Tables

**Table 1:** Demographic data of the study population.

	Group A	Group B	Total	*P* value	EF (95% CI)
Age (year)	37.2 ± 10.6	37.3 ± 11.5	37.4 ± 11.1	0.9	−0.01 (−0.32 to 0.30)
Weight (Kg)	74.1 ± 8.9	73.4 ± 11.8	73.8 ± 10.4	0.9	0.07 (−0.25 to 0.38)
Male/female	1.75	1.88	1.82	0.6	0.74 (0.37 to 1.10)

Group A: patients received morphine suppository.

Group B: patients received indomethacin suppository.

95% CI: 95% confidence interval.

EF: effect size.

**Table 2:** The mean decrease in the pain score during the first 20, 40, 60, and 90 minutes after the admission in groups A and B.

Duration	Group	Decrease in the pain score		
		Mean ± SD	*P* value	EF (95% CI)
0–20 minutes	Group A	5.46 ± 1.34	<0.001^*∗*^	0.73 (0.40 to 1.05)
	Group B	4.37 ± 1.63		

0–40 minutes	Group A	6.26 ± 1.62	0.3	0.16 (−0.18 to 0.45)
	Group B	6.04 ± 1.59		

0–60 minutes	Group A	6.27 ± 1.79	0.5	0.16 (−0.22 to 0.40)
	Group B	6.11 ± 1.66		

0–90 minutes	Group A	6.28 ± 1.75	0.4	0.13 (−0.18 to 0.44)
	Group B	6.07 ± 1.67		

Group A: patients received morphine suppository.

Group B: patients received indomethacin suppository.

95% CI: 95% confidence interval.

EF: effect size.

SD: standard deviation.

^*∗*^Statistically significant.

**Table 3:** The mean change in the systolic and diastolic blood pressure among the two groups during the first 20, 40, 60, and 90 minutes after the admission.

Duration	Group	Change in the systolic blood pressure			Change in the diastolic blood pressure		
		Mean ± SD	*P* value	EF (95% CI)	Mean ± SD	*P* value	EF (95% CI)
0–20 minutes	Group A	3.18 ± 3.62	0.1	0.16 (−0.9 to 0.53)	0.65 ± 2.34	0.3	0.15 (−0.16 to 0.46)
	Group B	2.16 ± 5.37			0.13 ± 4.29		

0–40 minutes	Group A	1.43 ± 3.33	0.2	−0.2 (−0.51 to 0.12)	0.71 ± 2.39	0.7	0.05 (−0.26 to 0.36)
	Group B	2.39 ± 5.98			0.52 ± 4.47		

0–60 minutes	Group A	1.79 ± 3.98	0.09	−0.28 (−0.59 to 0.04)	0.97 ± 2.81	0.4	0.12 (0.19 to 0.43)
	Group B	3.19 ± 5.94			0.52 ± 4.38		

0–90 minutes	Group A	1.64 ± 3.5	0.09	−0.29 (0.60 to 0.03)	0.92 ± 3.02	0.7	−0.05 (−0.36 to 0.26)
	Group B	3.03 ± 5.83			1.06 ± 2.39		

Group A: patients received morphine suppository.

Group B: patients received indomethacin suppository.

95% CI: 95% confidence interval.

EF: effect size.

SD: standard deviation.

**Table 4:** Comparison of adverse effects between the two study groups.

	Morphine group	Indomethacin group	*P* value	EF (95% CI)
Nausea	52.7%	47.3%	0.5	0.11 (−0.22 to 0.46)
Vomiting	49%	51%	0.7	−0.04 (−0.38 to 0.29)
Dizziness	43.3%	56.7%	0.4	−0.29 (−0.64 to 0.04)
Mouth dryness	48%	52%	0.4	−0.08 (−0.43 to 0.25)
Allergic reaction	49.7%	50.3%	0.5	−0.01 (−0.35 to 0.33)

95% CI: 95% confidence interval.

EF: effect size.
